# Efficacy of (R)-6-Adamantane-Derivatives of 1,3-Oxazinan-2-One and Piperidine-2,4-Dione in The Treatment of Mice Infected by the A/California/04/2009 influenza Virus

**DOI:** 10.32607/actanaturae.11020

**Published:** 2021

**Authors:** E. A. Glubokova, I. A. Leneva, N. P. Kartashova, I. N. Falynskova, R. M. Tikhov, N. Yu. Kuznetsov

**Affiliations:** I. Mechnikov Research Institute for Vaccines and Sera, Moscow, 105064 Russia; A. N. Nesmeyanov Institute of Organoelement compounds Russian Academy of Sciences, Moscow, 119991 Russia

**Keywords:** influenza virus, antiviral drugs, rimantadine, mouse model of influenza viral pneumonia

## Abstract

The World Health Organization (WHO) recommends antivirals as an additional line of defense against influenza. One of such drugs is rimantadine. However, most of the circulating strains of influenza A viruses are resistant to this drug. Thus, a search for analogs effective against rimantadine-resistant viruses is of the utmost importance. Here, we examined the efficiency of two adamantane azaheterocyclic rimantadine derivatives on a mouse model of pneumonia caused by the rimantadine-resistant influenza A virus /California/ 04/2009 (H1N1). BALB/c mice inoculated with the virus were treated with two doses (15 mg and 20 mg/kg a day) of tested analogs via oral administration for 5 days starting 4 hours before the infection. The efficacy was assessed by survival rate, mean day to death, weight loss, and viral titer in the lungs. Oral treatment with both compounds in both doses protected 60–100% of the animals, significantly increased the survival rate, and abolished weight loss. The treatments also inhibited virus titer in the lungs in comparison to the control group. This treatment was more effective compared to rimantadine at the same scheme and dosage. Moreover, the study of the sensitivity of the virus isolated from the lungs of the treated mice and grown in MDCK cells showed that no resistance had emerged during the 5 days of treatment with both compounds.

## INTRODUCTION

Influenza A viruses are a diverse group of respiratory pathogens that cause acute infections in humans, mammals, and birds [1]. Despite the availability of vaccines and antiviral drugs, influenza viruses cause annual epidemics and pandemics accounting for up to 650,000 deaths each year over the world, with up to 40,000 deaths in the United States alone [2]. In the past 10–15 years, from 27.3 to 47.2 million cases of acute respiratory viral infections have been registered annually, with the influenza infection responsible for 25–60% of all cases, depending on the intensity of the epidemics. The emergence of influenza pandemics, usually occurring every 20-30 years, is of particular concern. Along with the direct impact on public health, especially on populations from high-risk groups [2], infections lead to a huge, hard-to-measure, negative economic effect, as follows from the current COVID-19 pandemic. Vaccination is considered by the WHO as the mainstay in the prophylaxis of an influenza virus infection. However, due to the high and unpredictable variability of the influenza virus surface proteins, the composition of the vaccine is constantly changing depending on the antigenic structure of the circulating strains of influenza viruses. Therefore, the WHO, in addition to vaccination, recommends the use of small molecule antivirals that are especially important in a pandemic caused by new strains of the influenza A virus.

**Fig. 1:**
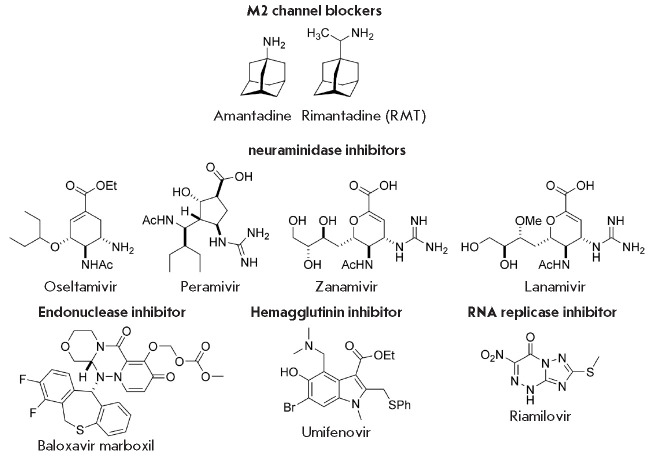
The structures of the drugs active against influenza viruses

Currently, there are two classes of anti-influenza drugs that have been approved worldwide [1, 3, 4]:** M2 channel blockers **– aminoadamantanes – amantadine and rimantadine (**RMT**) (*Fig. 1*) and **neuraminidase inhibitors **– oseltamivir, zanamivir, peramivir and lanamivir (only in Japan) (*Fig. 1*). M2 channel blockers belong to the first generation of antivirals effective against the influenza A virus. Although they have been successfully used for the treatment of influenza for more than 30 years [3, 4], their use has not been recommended since 2006 due to the widespread drug resistance of circulating strains [5]. The drug resistance has formed as a result of both evolutionary changes in the influenza virus and direct mutations during patient treatment with rimantadine and amantadine. Amantadine and **RMT **have a lower genetic barrier to drug resistance (1–2 passages) that has been shown in numerous experiments on animals or in cell cultures, and the drug resistance in humans can develop within 2–4 days after the start of treatment with these drugs [6]. The genetic basis of the resistance is mutations in gene 7 in the spliced second reading frame encoding the M2-protein and is associated with the replacement of amino acids at positions L26, V27, A30, S31 and G34 [7]. Mutation S31N (serine- arginine) is the most common case of resistance to aminoadamantanes in humans, avians and pigs [8]. Nevertheless, the unique and extensive experience in the successful clinical use of adamantane-type drugs worldwide leaves them in the arsenal of antiviral therapy as reserve drugs used to treat the appearing sensitive influenza strains that can be resistant to other influenza drugs: in particular, neuraminidase inhibitors. It should be noted that the emergence of oseltamivir-resistant strains has been continuously reported and was prevalent in the 2008–2009 seasonal influenza, when almost all circulating H1N1 strains had the H275Y mutation in the neuraminidase gene [9] while maintaining sensitivity to adamantanes.

As a result of efforts to overcome the existing resistance of influenza viruses to the first two classes of drugs, baloxavir marboxil, an endonuclease inhibitor has been elaborated, which is highly effective against various strains of influenza A and B viruses (approved in Japan, undergoing the last stage of trials in the USA) [10, 11, 12]. In addition, there are two drugs approved in Russia and China: umifenovir (“Arbidol”), which is an inhibitor of the fusion induced by hemagglutinin [13,14], as well as riamilovir (“Triazavirin”, Russia), an RNA-replicase inhibitor (*Fig. 1*).

To ensure reliable protection of the population in the face of an influenza epidemic, it is essential to have a set of antivirals acting through different mechanisms [15]. Unfortunately, there are currently no approved effective M2-blockers for the S31N virus. The influenza M2 channel is a highly conserved virus region, and, according to recent studies, experimental M2-blockers are quite sustained for resistance development [16]. Moreover, in the case of occurrence of such mutated strains, most of them [16, 17] do not remain in the viral population, suggesting that the elaboration of M2-blockers is a promising avenue.

**Scheme:**
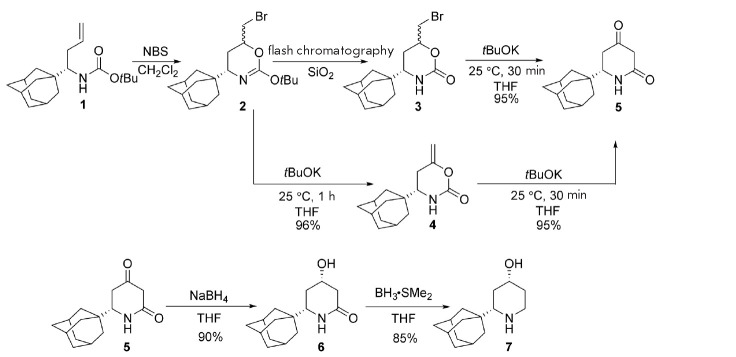
The structures of new adamantane derivatives active against rimantadine-resistant strains of the H1N1 influenza virus

Previously, we developed a convenient method for the synthesis of new enantiomerically pure 6-adamantyl derivatives of 1,3-oxazinan-2-ones and piperidines **3**–**7** from corresponding enantiomeric homoallylamines **1** (*Scheme*). The key steps in the process were bromocyclocarbamation (**1 **into **2 **and **3**), dehydrobromination by *t*BuOK (**2 **into **4**), and enolate-isocyanate rearrangement (**4 **into **5**). The last two reactions are “one pot” in the case of bromide **3**. Diketone **5 **was then reduced stepwise to 4-hydroxylactam **6 **and to 4-hydroxypiperidine** 7**. The obtained compounds **3**–**7 **were found to inhibit *in vitro *replication of the pandemic strains A/California/7/2009 and A/IIV-Orenburg/29-L/2016 bearing the S31N mutation [18]. In each pair of enantiomers, (*R*)-isomers (asymmetric center at the adamantyl group) of **3**–**5 **and **7 **inhibited *in vitro *replication of the influenza viruses most effectively (*Scheme*, *Table 1*).

**Table 1:** Inhibition of influenza A H1N1 virus replication by inhibitors 3–7 *in vitro*

Virus strain	IC_50_, μМ			
	3	4	5	7
A/California/7/2009 (H1N1)	11.3	8.1	20.6	18.4
A/IIV-Orenburg/29-L/2016 (H1N1)	20.1	7.7	27.1	17.7

Since the *in vitro *inhibitory activity of the compounds was quite promising, their effectiveness *in vivo* had to be tested. However, compound **3 **was excluded from the study due to its low solubility in aqueous solutions, as well as compound **7, **which was rather difficult to synthesize in diastereomerically pure form. Thus, (*R*)-6-adamantyl derivatives of 1,3-oxazinan-2-one **4** and piperidin-2,4-dione **5 **were selected, due to their simplicity of synthesis and acceptable solubility in aqueous solutions. Evaluation of the activity of the compounds **4**, **5 **was carried out on a mouse model of pneumonia induced by the rimantadine-resistant influenza virus A/California/04/09 (H1N1).

## EXPERIMENTAL PROCEDURES

**Compounds and their preparation**

(*R*)*-*isomers of compounds **4 **and **5 **were synthesized from the corresponding (*R*)*-N*-Boc-derivative of adamantyl homoallylamine **1**, according to the procedures described in [18]. For each experiment, freshly made solutions of compounds **4**, **5 **and **RMT **in 50% DMSO were used. The studied solutions were administered orally to mice in a volume of 200 ѓKl, and the animals were treated by compounds **4**, **5**, and **RMT **in doses of 15 and 20 mg/kg/day.

**Cells and viruses**

MDCK cells were grown in a modified Eagle’s medium (MEM; CellGro, Manassas, VA) supplemented with 10% FBS and 5 mM L-glutamine, 25 mM HEPES, 100 U/ml penicillin, 100 μg/ml streptomycin sulfate, and 100 μg/ml kanamycin sulfate in a humidified atmosphere of 5% CO_2_. The influenza A/California/ 04/2009 (H1N1) virus was provided by the WHO National Influenza Centre of Russia (St. Petersburg, Russia) and mouse-adapted by three lung-to-lung passages. The virus stock grown in the allantoic cavity of 9-day-old embryonated chicken eggs for 48 h at 37°C was used to modulate the influenza infection in the animals according to the conventional technique [19].

**Animals**

Inbred female mice (12–14 g) were obtained from the Andreevka Research Centre for Biomedical Technology (Moscow Region). Animal maintenance and care were performed in accordance with the Guide for the Care and Use of Laboratory Animals. The mice were fed with briquetted feed following the approved standards. All studies were approved by the I.I. Mechnikov Research Institute of Vaccines and Sera Committee on the Ethics of Animal Experiments.

**Assessment of drug efficacy in a mouse model**

The mice were group-housed in cages and used at a quantity of 8–13 mice per treatment group. On the day of experiment, the mice were weighed and then infected intranasally under light anesthesia. In the first series of experiments, a high infection dose of 10 MLD_50_ (mouse lethal dose of 50) was used, corresponding to 4.5 lgTCID50 (tissue cytopathic infectious dose of 50); in the second series of experiments – a low dose of MLD90, corresponding to 4.0 lgTCID50. Compounds **4**,** 5 **and **RMT **(control drug) were administered by oral gavage in a 0.2 ml volume to every animal 4 h before and after infection, and the treatment continued for 5 days twice daily. The placebo was administered in parallel with the antiviral treatments (PBS in experiment 1 or 50% DMSO in experiment 2). The survival rate and weight change were observed for 16 days after virus inoculation. The animals that showed signs of severe disease and weight loss of 30% were humanely euthanized. The efficacy of the compounds in the mouse model of influenza pneumonia was estimated by the following criteria: survival rate; mean day to death (MDD); weight loss and viral titer reduction in the lungs in the treated animal groups compared to the control. MDD was calculated by the following formula

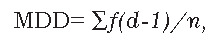



where *f *is the number of dead mice on day *d *(survivors on day 16 were included in *f *for that day) and *n* is the number of mice in the group. The weight loss or gain was calculated for each mouse as a percentage of its weight on day 0 before virus inoculation. The weight of an animal before inoculation was considered to be 100%. For all the mice in one group, an average value of their weight loss and gain was calculated. Four days after inoculation, three mice from each group were sacrificed: their lungs were removed under sterile conditions to be thoroughly rinsed with 0.01 M sterile PBS, homogenized, and suspended in 1 mL of cold PBS. After separation of the cell debris by centrifugation at 2000 *g *for 10 min, the supernatant was used to determine the viral titer in the MDCK cell culture by the generally accepted method. Virus titers in mouse lungs were calculated as the mean lgTCID_50_/mL ± } SD.

Statistical processing of the data was carried out using the log-rank Mantel-Cox test in the Statistica 8.0 program with the *p * < 0.05 value considered a statistically significant difference from the control.

**Antiviral activity by cell-based ELISA assay**

Stock-solutions (1 mg/ml) of samples and **RMT** prepared in DMCO were used to prepare final concentrations. MDCK cells were seeded in 96-well plates (3,000 cells/well, “Costar”) and grown as a confluent monolayer, washed twice with serum-free MEM, and overlaid with MEM (100 μl) containing 2.5 μg/ml *N*-tosyl-L-phenylalanine chloromethyl ketone (TPCK)-treated trypsin (Sigma-Aldrich) with a final concentration range of 1–0 •µg/mL. After incubation for 2 h at 37°C, 100 μl of the virus isolated from the lungs of the treated animals containing approximately 0.1 PFU/cell was added to all wells, except the uninfected control cells. After a 24-hour incubation period, the cells were washed and fixed by adding 50 μl of cold 80% acetone in PBS. Viral expression was measured by ELISA, as previously described. For a point in the experiment, four wells of a plate were used and each value represented a mean calculated from three independent experiments.

**Sequence analysis of the M gene**

Identification of the molecular marker of drug resistance was carried out by sequencing of the *M2* gene of the influenza A/California/04/2009 (H1N1) pdm09 virus that was used to infect the animals. Total RNA was extracted using a RIBO-prep nucleic acid extraction kit (AmpliSens, CRIE, Russia). A REVERTA-L reagents kit (AmpliSens, CRIE, Russia) and 5•Lagcaaaagcagg primer were used for reverse- RNA transcription. Amplification of viral cDNA was conducted using such primers as M 1F agcaaaagcaggtagatgtt; M 1027R agtagaaacaaggtagttt on a Tercyc thermocycler (DNA-Technology, Russia). Sequencing reactions of overlapping PCR products were conducted with the same primers used for amplification with an ABI PRISM Big DyeTM v.3.1 Cycle Sequencing Reaction Kit according to the manufacturer’s instructions on an ABI-3100 PRIZMTM Genetic Analyzer (Applied Biosystems, USA). All sequences were assembled with the Lasergene version 10.1 package (DNASTAR Inc, USA).

## RESULTS AND DISCUSSION

**The efficacy of compounds 4 and 5 at a dose of 20 mg/kg/day on a murine model of viral pneumonia induced by a high dose of the rimantadineresistant influenza A/California/04/2009 virus**

Preliminary experiments showed that the administration of the compounds under study in doses of up to 60 mg/kg/day according to the schemes used in the subsequent treatment of intact mice did not cause weight loss and mortality in any of the animals. To further study the effectiveness of compounds **4 **and **5 **in comparison with RMT, a dose of 20 mg/kg was chosen as an optimal dose for studying the effectiveness of RMT in mice [3].

**Fig. 2:**
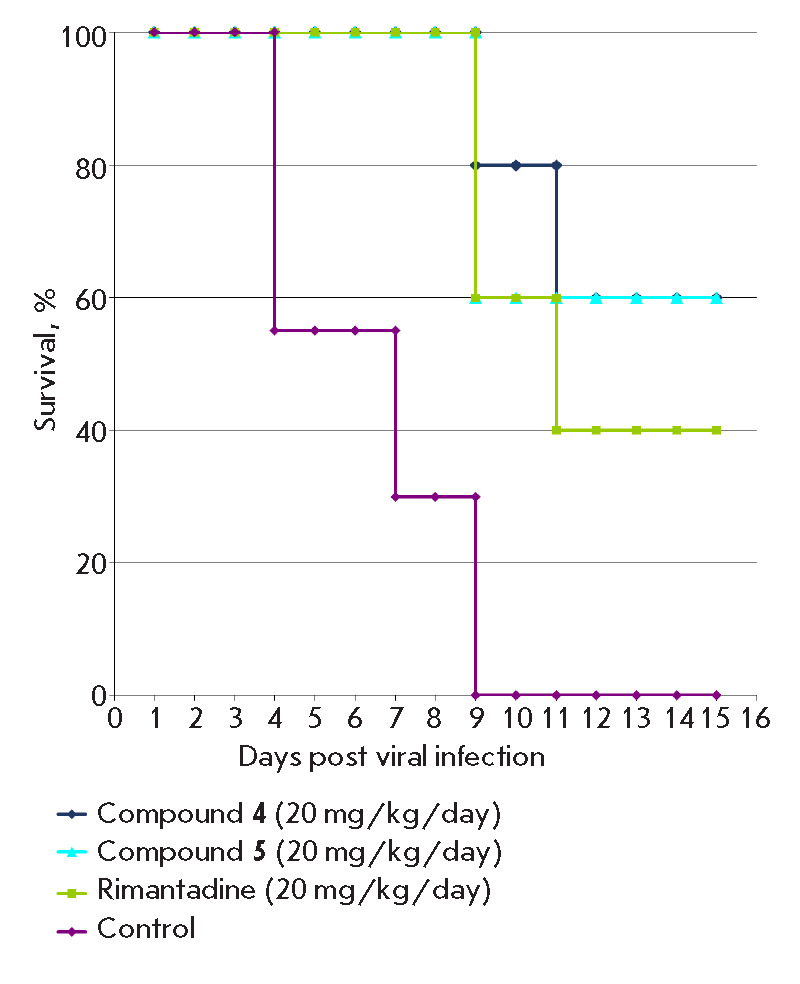
Survival rates of mice treated with compounds **4**,** 5 **in a murine model of influenza pneumonia induced by a high dose of the virus

**Fig. 3:**
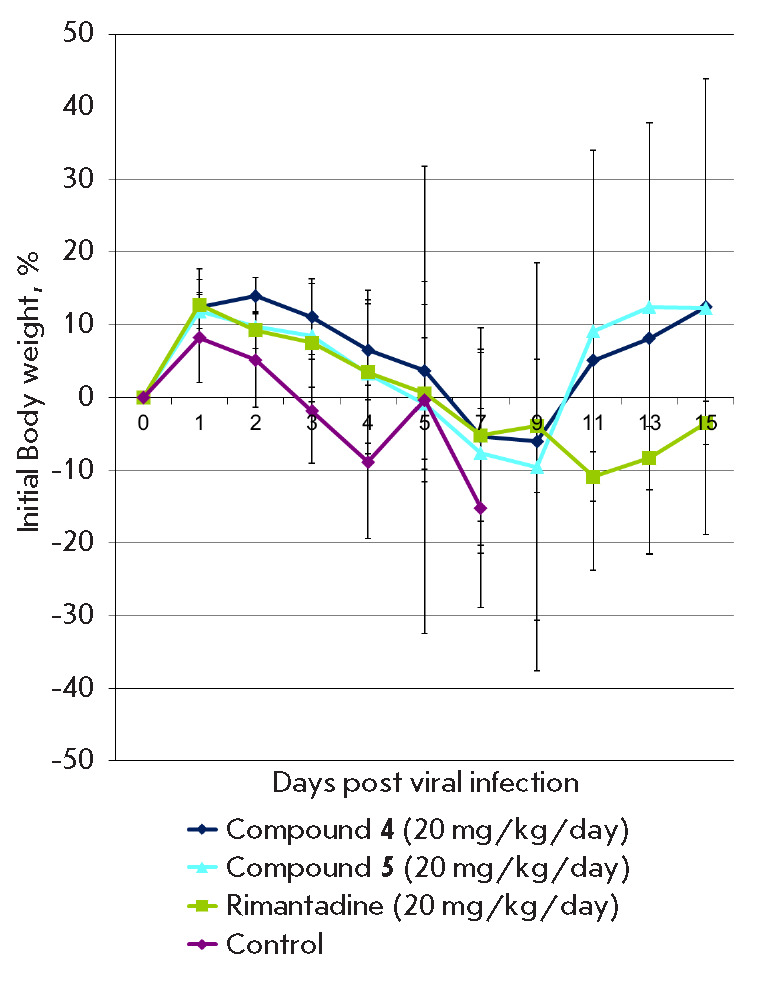
Change in the body weight of mice during treatment with compounds **4**, **5 **in a murine model of influenza pneumonia induced by a high dose of the virus

In the control group of infected animals not receiving any treatment, cases of death were observed starting from day 7 and mortality reached 100% by day 9: the mean day to death (MDD) in this group was 5.1 days. The loss of body weight in the control began from the second day after infection and reached its maximum value (18%) by day 5. Compounds **4 **and **5 **were equipotent, protecting 60% of the animals on the 15^th^ day of observation. Treatment of the mice with **4 **and **5 **was more effective than with **RMT **at the same dose, which provided protection to 40% of the animals. The MDD was 10.1 days for **RMT**, while for **4 **and **5 **it was more than 12 days. In addition, in the groups treated with all tested adamantanes (**4**, **5**, **RMT**), the weight loss was less significant than in the control group (*Fig. 2*, *Fig. 3*, *Table 2*).

**Table 2:** Efficacy of oral treatment with compounds 4 and 5 in a murine model of influenza pneumonia induced by a high dose of the influenza A/California/04/2009 (H1N1) pdm09 virus

Dose, mg/kg/ day	Survival		Protection from mortality, %	MDD, days
	Alive/ Total	Mortality, %		
Compound 4				
20	3/5^a^	40	60	12.6
Compound 5				
20	3/5^b^	40	60	12.2
RМТ				
20	2/5^c^	60	40	10.1
Virus control				
	0/10	100		5.1

^a^ – (р = 0.003198);

^b^ – (р = 0.003198);

^c^ – (р = 0.031863).

**Determination of the efficacy of compounds 4 and 5 at doses of 15 and 20 mg/kg/day on a mouse model of pneumonia induced by a low dose of the rimantadineresistant influenza virus A/California/04/2009**

To identify the differences in the actions of compounds** 4 **and **5**, in subsequent experiments the viral inoculation dose was reduced and two doses of 20 and 15 mg/kg/day of the compounds were selected

**Fig. 4:**
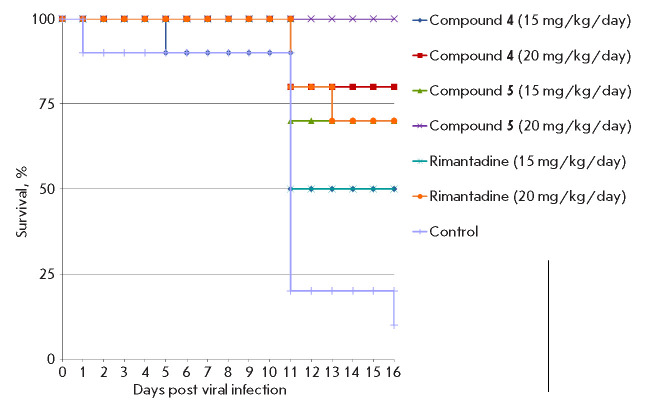
Survival of mice in a model of influenza pneumonia induced by a low dose of the virus

the control group of non-treated infected mice, death of animals by the 16th day of observation reached 90% and MDD in this group was 10 days (*Fig. 4*, *Table 3*). The oral administration of compound** 4 **in a dose of 15 mg/kg/day did not have a statistically significant effect on the survival rate;mortality in these groups was 50% (*Fig. 4*, *Table 2*). An increase of the dose to 20 mg/kg/day led to a significant decrease in mortality, to 20%. Compound **5 **was more effective – with treatment at a dose of 15 mg/kg/day the mortality rate was 30%, and a dose of 20 mg/kg/day fully protected the animals from death.

**Table 3:** Efficacy of oral treatment with compounds 4 and 5 in a murine model of influenza pneumonia induced by a low dose of the influenza A/California/04/2009 (H1N1)pdm09 virus

Dose, mg/kg/day	Survival		Protection from mortality, %	MDD, days	Viral titre, lg ТCID_50_
	Alive/Total	Mortality, %			
	Compound 4				
15	5/10^a^	50	40	12.4	4.5 ± 0.5
20	8/10^b^	20	70	14	1.16 ± 1.6
	Compound 5				
15	7/10^c^	30	60	13.5	2.5 ± 2.3
20	10/10^d^	0	100	> 16	2.6 ± 2.3
	RМТ				
20	7/10^e^	30	60	13.7	1.3 ± 0.3
	Virus control				
-	1/9	90	-	10	6.1 ± 0.3

^a^ – (р = 0.075134);

^b^ – (р = 0.001106);

^c^ – (р = 0.007137);

^d^ – (р = 0.000000168);

^e^ – (р = 0.007137).

**Fig. 5:**
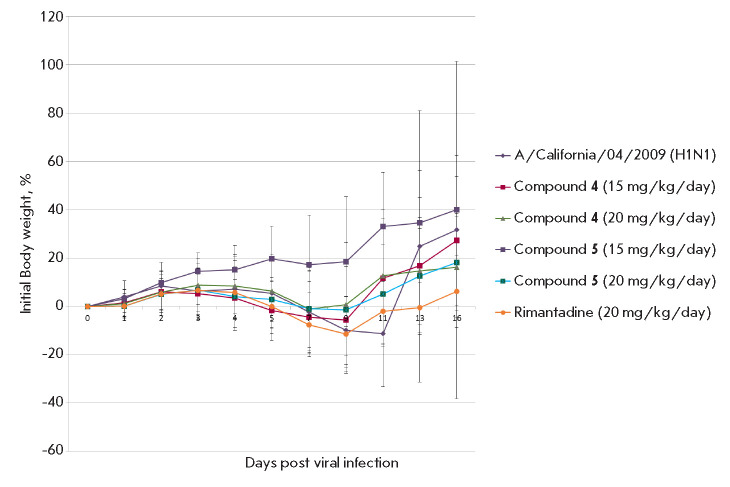
Change in the body weight of mice in a model of influenza pneumonia induced by a low dose of the virus

In the control group, body weight loss was observed starting from the 3^rd^ day after the viral infection, reaching 11% on average by the 11th day. Survival data was confirmed by the most important criterion for the severity of the disease – weight loss. In the groups treated with compound **5 **in both studied doses and with compound **4 **at a dose of 20 mg/kg/day, a decrease in body weight was not observed (*Fig. 5*). Treatment with** RMT **at a dose of 20 mg/kg/day led to a higher level of mortality (30%) and weight loss compared to the mice treated with the same dose of compounds **4 **and **5 **that correlated with the survival data.

The observed greater animal survival rate in the second series of experiments evidently was due to the reduced virus dose, since the effectiveness of the antiviral drug was inversely proportional to the dose of infection, as well as to the fact that for the initial screening of the compounds in the first experiment, the groups including a smaller number of animals were the ones studied. A dose-dependent increase in the effectiveness of the tested compounds was also observed. On the whole, the obtained data indicate a virus-specific effect of the studied compounds.

**The effect of treatment with RMT and compounds 4, 5 in various doses on the viral titer in the lungs of a mouse model of pneumonia induced by the rimantadine-resistant influenza virus A/California/04/2009**

The data on the increased survival rate were confirmed by a virological method. The viral titer reflects the replication of the virus in the lungs, its higher value corresponding to more severe pathological changes in the lungs. The highest viral titer (6.1 ± 0.3 lg TCID50) measured was in the control group. The smallest suppression of the viral titer was observed during treatment with compound **4 **at a dose of 15 mg/kg/day (4.5 ± 0.5 lg TCID50). An increase in the dose of compound** 4 **to 20 mg/kg/day, as well as treatment with compound **5 **at both doses, significantly inhibited the replication of the virus, reducing the titer by 2.4–4.9 lg TCID50, which corresponded to the clinical parameters of treatment efficiency obtained for these compounds. It is also important to note a significant suppression of virus replication in the lungs when treated with **RMT**. Although the mortality rate for **RMT **applied at a dose of 20 mg/kg/day in both series of experiments was higher than that with compounds **4 **and **5 **at the same dose, it was statistically significantly lower compared to the group of infected untreated animals. Since previously no **RMT **activity had been observed in the cell culture with the rimantadine-resistant influenza virus A/California/04/2009(H1N1), data demonstrating such activity in experiments with mice was somewhat unexpected. However, it must be stressed that the data obtained *in vivo *more adequately characterize antiviral activity, since they account for such features as compound bioavailability, toxicity, and pharmacokinetics directly in the body. Often, the drug concentrations reached in blood plasma can significantly exceed the necessary concentrations to suppress antiviral activity in *in vitro *experiments. This may explain the efficacy of the drugs in respect to viruses resistant to them. A similar effect was noted in the study of the efficacy of oseltamivir in ferrets [20], where oseltamivir was effective not only against oseltamivir-sensitive, but also against oseltamivir-resistant H1N1 influenza viruses with the H274Y mutation, though to a lesser degree. These data are also in agreement with the clinical studies that showed the efficacy of oseltamivir during the 2008–2009 epidemic season, when the oseltamivir- resistant strain H1N1 (H274Y) was in circulation: however, this efficacy was lower than that of another neuraminidase inhibitor, zanamivir, to which the virus strain was also sensitive [21]. Very similar results were reported by the authors of [22], where **RMT **efficacy in the treatment of influenza during seasons with the circulation of the rimantadine-resistant strain A/California/ 04/2009 (H1N1) was observed. However, the efficacy of such treatment was lower compared to that of oseltamivir, which was used in the same studies.

**Sequence analysis of the mouseadapted rimantadine-resistant influenza A/California/04/2009 virus**

influenza virus A/California/04/2009pdm (H1N1) has a mutation, S31N, in the M2 protein, which is a molecular genetic marker of resistance to adamantanes. Although in our experiments we showed that treatment with novel adamantanes was more effective than treatment with **RMT**, the fact that **RMT **itself reduced animal mortality, weight loss, and virus replication in the lungs of mice infected by the rimantadine-resistant influenza A/California/04/2009pdm (H1N1) virus was notable. In actuality, the origin strain of A/California/ 04/2009pdm (H1N1) is not lethal for mice; therefore, in our experiments, we used a virus adapted to mice by passaging it into the lungs of the animals. We assumed that the mutation responsible for resistance to** RMT **could be lost in the process of adaptation. To verify this assumption, sequencing of gene 7 encoding the M2 protein of the mouse-adapted virus was performed. The nucleotide sequence of the 7^th^ gene found showed that, in our mouse-adapted strain, the S31N mutation responsible for virus resistance to rimantadine was, indeed, preserved.

**The possibility of occurrence of resistance to compounds 4 and 5 in the course of their intake**

**Fig. 6:**
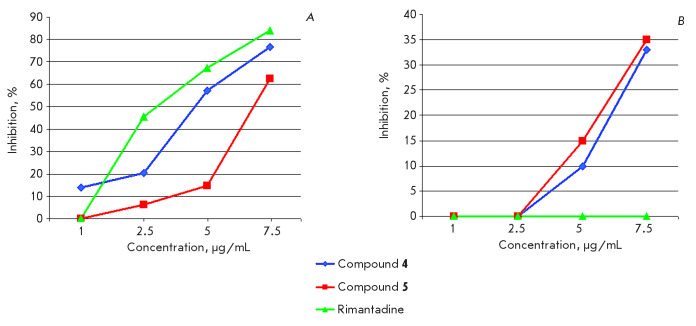
Antiviral activity of compounds **4**, **5**, and **RMT **in a MDCK cell culture against influenza A / Aichi / 2/68 (H3N2) (*A*) and A / California / 04/09 (H1N1) viruses isolated from the lungs of treated animals (*B*)

Another important aspect in the development of antiviral drugs is that drug resistance occurs during infection treatment. As was mentioned before influenza A viruses develop resistance to adamantanes in a cell culture and in animals just after 2–3 passages; in a human population, such strains can appear within 2–4 days after the start of treatment [4, 5, 6]. To study the possible emergence of resistane to compounds **4 **and** 5**, the viruses were isolated from the lungs of treated (with both compounds or **RMT) **mice on the 4^th^ day post-infection and their sensitivity studied in MDCK cells. For comparison, influenza A/Aichi/2/68(H3N2) virus sensitive to **RMT **was used (*Fig. 6*). It can be seen that both compounds **4**, **5, **and **RMT **were active against this virus. At the same time, the viruses isolated from the lungs of the mice infected with the rimantadine-resistant influenza A/California/04/2009pdm (H1N1) virus and treated with compounds **4 **and **5 **remained sensitive to them, which is an indication that no resistance to these compounds had developed during their repeated application. The results are in accordance with literature data demonstrating that, unlike **RMT**, no resistance to S31N-M2-blockers occurs in the course of treatment [16].

## CONCLUSIONS

According to the previously developed convenient and efficient method, the (*R*)-isomers of 6-(1-adamantyl)- 1,3-oxazinan-2-one **4 **and 6-(1-adamantyl) piperidin-2,4-dione **5 **were synthesized in preparative gram-scale quantities to study the antiviral activity of a murine model of viral pneumonia induced by the influenza A virus. Both compounds, administered orally in doses of 15 and 20 mg/kg/day, protected mice, significantly reducing animal mortality, weight loss, virus replication in the lungs of the animals, and they increased survival of the animals (mean day to death). The treatment of mice with compounds **4 **and **5 **was more effective than treatment with the comparative drug rimantadine at the same doses and scheme. It is noteworthy that application of these novel adamantanes for 5 days did not lead to the development of resistance to them. The compounds effectively inhibit the replication of influenza A viruses, including rimantadin- resistant strains. The synthetic scheme of these adamantane derivatives is simple and contains easily available compounds. It is our hope that directed modification of the structures of adamantyl (hydroxylation) and heterocyclic (substitution in the 4^th^ position of compounds **4 **and **5**) fragments of these compounds would further enhance their antiviral activity and shed light on how they block the M2 channel. Given the abovesaid, the studied heterocyclic adamantanes are promising for the development of new therapeutic agents for the treatment of the influenza A infection.

## Abbreviations

MDD: mean day to death
IC50: 50% inhibitory concentration
TCID50: 50% tissue cytopathic infective dose
MLD: mouse lethal dose
PSB: Phosphate buffered saline
MEM: Minimum Essential Medium
MDCK: Madin-Darby canine kidney
WHO: World Health Organisation
ELISA: enzyme-linked immunosorbent assay
DMSO: Dimethyl sulfoxide
RMT: rimantadine
pdm: pandemic
